# Computational Analysis of the Immune Infiltration Pattern and Candidate Diagnostic Biomarkers in Lumbar Disc Herniation

**DOI:** 10.3389/fnmol.2022.846554

**Published:** 2022-04-21

**Authors:** Kai Li, Shijue Li, Haojie Zhang, Di Lei, Wai Leung Ambrose Lo, Minghui Ding

**Affiliations:** Department of Rehabilitation Medicine, The First Affiliated Hospital, Sun Yat-sen University, Guangzhou, China

**Keywords:** lumbar disc herniation, GEO dataset, diagnostic biomarkers, immune cell infiltration, pain

## Abstract

**Objectives:**

Lumbar disc herniation (LDH) is a musculoskeletal disease that contributes to low back pain, sciatica, and movement disorder. Existing studies have suggested that the immune environment factors are the primary contributions to LDH. However, its etiology remains unknown. We sought to identify the potential diagnostic biomarkers and analyze the immune infiltration pattern in LDH.

**Methods:**

The whole-blood gene expression level profiles of GSE124272 and GSE150408 were downloaded from the Gene Expression Omnibus (GEO) database, including that of 25 patients with LDH and 25 healthy volunteers. After merging the two microarray datasets, Differentially Expressed Genes (DEGs) were screened, and a functional correlation analysis was performed. The Least Absolute Shrinkage and Selection Operator (LASSO) logistic regression algorithm and support vector machine recursive feature elimination (SVM-RFE) were applied to identify diagnostic biomarkers by a cross-validation method. Then, the GSE42611 dataset was used as a validation dataset to detect the expression level of these diagnostic biomarkers in the nucleus pulposus and evaluate their accuracy. The hub genes in the network were identified by the CIBERSORT tool and the Weighted Gene Coexpression Network Analysis (WGCNA). A Spearman correlation analysis between diagnostic markers and infiltrating immune cells was conducted to further illustrate the molecular immune mechanism of LDH.

**Results:**

The azurophil granule and the systemic lupus erythematosus pathway were significantly different between the healthy group and the LDH group after gene enrichment analysis. The XLOC_l2_012836, lnc-FGD3-1, and scavenger receptor class A member 5 were correlated with the immune cell infiltration in various degrees. In addition, five hub genes that correlated with LDH were identified, including AQP9, SIRPB2, SLC16A3, LILRB3, and HSPA6.

**Conclusion:**

The XLOC_l2_012836, lnc-FGD3-1, and SCARA5 might be adopted for the early diagnosis of LDH. The five identified hub genes might have similar pathological mechanisms that contribute to the degeneration of the lumbar disc. The identified hub genes and immune infiltrating pattern extend the knowledge on the potential functioning mechanisms, which offer guidance for the development of therapeutic targets of LDH.

## Introduction

Low back pain (LBP) affects approximately 1.71 billion people worldwide and is the main contributor to the global burden of musculoskeletal conditions (Cieza et al., 2021). Lumbar disc herniation (LDH) is a pathology that causes LBP, sciatica, and movement disorders (Clark and Horton, 2018). LDH causes LBP symptoms by mechanical compression, chemical radiculitis, and autoimmunity. However, the exact pathological immune mechanism of LDH remains unknown. A study illustrated that immune environment factors are a contributing factor to inflammation and pain exacerbation for patients with LDH. Until now, no effective medical therapy has been available for LDH. Therefore, identifying the vital biomarkers and revealing the relationship is of great significance to developing effective treatment strategies for patients with LDH.

The intervertebral disc is a fibrocartilage that connects two adjacent vertebrae. The disc consists of the outer annulus fibrosus (AF), inner nucleus pulposus (NP), and cartilage endplate (CEP). A previous study reported that intervertebral disc degeneration and aging are crucial factors that contribute to the dehydration of the NP, consequently weakening the AF (Gorth et al., 2019). As the cartilage endplate becomes weak, fissures will appear in the AF, and its shock-absorbing ability will be limited and will eventually contribute to LDH. An increasing number of studies suggest that the immune environment plays an important role in the occurrence and deterioration of LDH. Sun et al. (2020) showed that the damage of the blood-NP barrier (BNB), an immune privilege of the intervertebral disc, plays a significant role in the whole process of LDH. Therefore, identifying the differential gene expression will assist the clarification of the molecular mechanism that underpins LDH and develops new immunotherapy targets. To date, only a few studies that have investigated the molecular immune mechanism of the development of LDH were found.

## Materials and Methods

### Selecting and Preprocessing Data

In the Gene Expression Omnibus (GEO) database^1^, intervertebral disc degeneration and lumbar disc herniation were set as the retrieval condition. Based on the sample size and retrieval condition of the lumbar disc, the datasets GSE124272 (Wang et al., 2019) and GSE150408 (Wang et al., 2021) were selected. Whole-blood RNA-seq transcriptome data were obtained from eight patients in the GSE124272 dataset and 17 patients in the GSE150408 dataset. In addition, the patients with treatment were excluded from the transcriptome data in the GSE150408 dataset. Healthy group data were obtained from eight healthy volunteers in the GSE124272 dataset and 17 healthy volunteers in the GSE150408 dataset.

**FIGURE 1:**
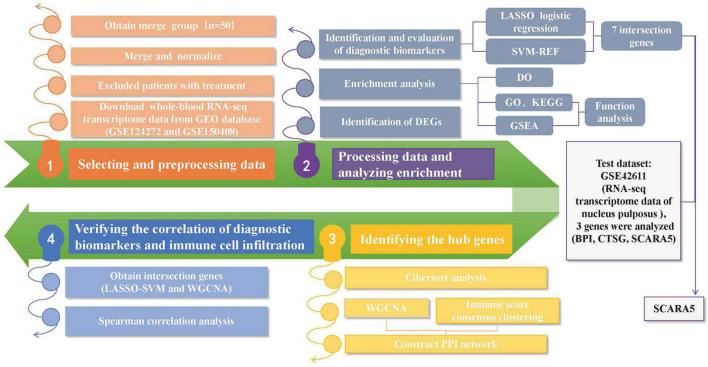
A flow-process diagram showing the analysis steps in this study (GEO: Gene Expression Omnibus; DEGs: Differentially Expressed Genes; ROC: Receiver operating characteristic; LASSO: Least Absolute Shrinkage and Selection Operator; SVM-RFE: the support vector machine recursive feature elimination; GO: Gene Ontology; KEGG: Kyoto Encyclopedia of Genes and Genomes; DO: Disease Ontology; GSEA: Gene Set Enrichment Analysis; WGCNA: Weighted Gene Coexpression Network Analysis; PPI: Protein-protein interaction).

The “R” software (R v4.1.1)^2^ was adopted for the analysis. The Practical Extraction and Report Language (Perl)^3^ was applied to accurately handle the text formats that were required for the R package analysis. Figure 1 shows the analysis steps in this study. Two gene expression matrices were merged, and the inter-batch differences were removed for next-stage analysis. The “ggplot2” package (Skidmore et al., 2016) was applied to draw the two-dimensional PCA cluster plot and to visualize the effect after data normalization.

### Processing Data and Analyzing Enrichment

The “limma” package (Ritchie et al., 2015) was adopted to screen differentially expressed genes (DEGs), and the “ConsensusClusterPlus” package (Wilkerson and Hayes, 2010) was applied to cluster the LDH dataset into different groups on account of the expression similarity. Then, the “pheatmap” package and the “ggplot2” package (Steenwyk and Rokas, 2021) were applied to visualize the expression of DEGs. The selection criteria were |log2 FC| > 1, and false discovery rate (FDR) was < 0.05.

The “clusterProfiler,” (Yu et al., 2012) “org.Hs.eg.db,” “enrichplot,” and “ggplot2” packages were applied to perform Gene Ontology (GO) (Gene Ontology Consortium, 2015) and Kyoto Encyclopedia of Genes and Genomes (KEGG) (Kanehisa and Goto, 2000; Kanehisa et al., 2017). Then, the “GSEABase” package and the “DOSE” were applied to analyze Disease Ontology (DO) (Yu et al., 2015) enrichment on DEGs. The GO analysis consisted of biological process (BP), molecular function (MF), and cellular component (CC). The KEGG analysis was adopted to identify the pathways of biological molecular interaction. The DO analysis was applied to explore the similarity of diseases. The level of FDR < 0.25 and *p* < 0.05 were chosen to find out the significant function enrichment.

The “clusterProfiler” package was adopted to perform Gene Set Enrichment Analysis (GSEA) (Reimand et al., 2019) on the gene expression matrix. The “c2.cp.kegg.v7.0.symbols.gmt” and “c5.go.v7.4.symbols.gmt” were applied to analyze significant enrichment between the healthy group and LDH group. Subsequently, the results were illustrated in the enrichment plot by applying the “enrichplot” package. The GSEA is another enrichment analysis to identify significant biological changes in the microarray datasets. Net enrichment score (NES), gene ratio, and *p*-value in the GSEA analysis were applied to verify the GO and KEGG enrichment results.

### Screening and Verifying Diagnostic Biomarkers

The “glmnet” package was applied to analyze DEGs by the application of the Least Absolute Shrinkage and Selection Operator (LASSO) logistic regression algorithm. The feature sorting method of support vector machine recursive feature elimination (SVM-RFE) (Zhang et al., 2018) was conducted to improve the accuracy of identifying the diagnostic biomarkers by analyzing appropriate datasets selected by the LASSO algorithm to obtain biomarkers. The “e1071” package, “kernlab” package, and “caret” package were applied to identify DEGs from whole-blood gene expression profiles by applying SVM-RFE. The “VennDiagram” package was applied to draw a Venn plot which shows the screened intersection genes after using the LASSO algorithm and SVM-RFE method to analyze the gene expression profiles. The GSE42611 dataset was used as a validation dataset to detect the gene expression level of intersection genes in the nucleus pulposus. The “pROC” package was adopted to draw receiver operating characteristic (ROC) curves (Mandrekar, 2010), calculate AUC, and evaluate values of diagnostic biomarkers.

### Identifying the Hub Genes

The “CIBERSORT” method (Newman et al., 2015) was applied to analyze the level of immune cell infiltration. Then, the “e1071” package was adopted to calculate the relative ratio of immune cells and immunity score (Chen et al., 2018). Moreover, the “corrplot” package was used to draw the correlation graph of 22 types of infiltrating immune cells. Due to the sample size of DEGs, the merged group was chosen to analyze and filter out the low expression data. Based on the gene difference analysis, the “ConsensusClusterPlus” package was applied to cluster the “merge” data set into different groups for gene expression similarity. Then, the “ggpubr” package was applied to analyze the immune infiltration of DEGs between the healthy group and the LDH group. Besides, the “ggplot2” package was adopted to draw a boxplot to show the difference in infiltrating immune cells.

The immune cell infiltration-related genes were identified by the Weighted Gene Coexpression Network Analysis (WGCNA) (Langfelder and Horvath, 2008), revealing the correlation between immune cell infiltration-related genes and exploring the phenotype and hub genes in the network. Total samples were clustered by average linkage and Pearson correlation value. β = 4 (scale free *R*^2^ = 0.9) was chosen to construct a scale-free network (Figure 5C). Then, a hierarchical clustering tree was constructed by the dynamic hybrid cutting technology to construct gene modules (minimum gene number of gene modules is 50). Branches represent a series of genes with similar expression data, and each leaf represents a gene in the tree (Figure 5D). In addition, six modules (Figure 5E) were built into the analysis. A heatmap was used to show the gene expression in six modules and two groups. Afterward, cluster analysis was carried out on gene modules and the modules were merged into a new dynamic tree. Gene significance (GS) and module membership (MM) were calculated. The relationship between gene expression and sample trait (including immune cell infiltration score) was determined. Lastly, the “VennDiagram” package (Chen and Boutros, 2011) was used to draw a Venn plot and show the intersection of DEGs and gene modules. Intersection genes were analyzed using the online tool STRING^4^ to construct a protein-protein interaction (PPI) network (Miryala et al., 2018), and Cytoscape software (Reimand et al., 2019) was employed to investigate the interaction and identify the hub genes.

### Verifying the Correlation of Diagnostic Biomarkers and Immune Cell Infiltration

The “corrplot” package was adopted to analyze the correlation of the 22 types of immune cells. In addition, intersection genes were filtered from the genes of LASSO logistic regression and significant gene modules. The “VennDiagram” package was applied to draw a Venn plot and show the intersection genes. The “ggpubr” package and the “ggExtra” package were applied to perform Spearman correlation analysis on diagnostic markers and infiltrating immune cells. Then, a lollipop drawing was applied to visualize the analysis results.

## Results

### Selecting and Preprocessing Data

**FIGURE 2:**
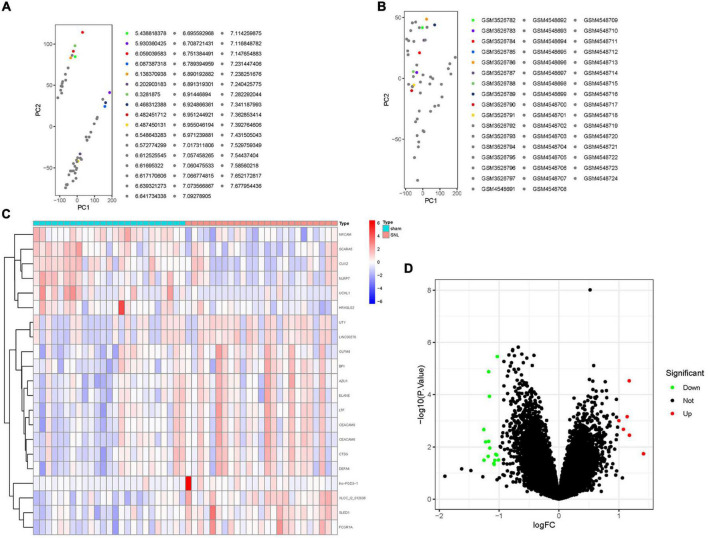
The distribution of RNA in lumbar disc herniation (LDH) after merging the GSE124272 and the GSE150408 datasets. **(A)** In the two-dimensional PCA cluster plot of the merged dataset before normalization, with each point representing a sample. **(B)** In the two-dimensional PCA cluster plot of the merged dataset after normalization, with each point representing a sample. **(C)** The expression level of RNA in LDH. The higher the level of expression, the darker the color (red represents upregulated, green represents downregulated). The tree on the left showed the clustering results of significant RNA in different samples. The grid on the right indicated the groups (red represented normal group and blue represented LDH group). **(D)** Volcano map of differentially expressed genes (DEGs); red represents upregulated differentially expressed genes (DGEs), gray represents no significant DGEs, and green represents downregulated DGEs.

The inter-batch differences between GSE124272 and GSE150408 datasets were eliminated after the two datasets merged. The clustering of the two datasets was evenly distributed after data normalization (Figures 2A,B), indicating a reliable sample source.

### Processing Data and Analyzing Enrichment

A total of 21 DEGs between the healthy and LDH groups were identified from the merging gene expression matrix. Figures 2C,D illustrate the expression of DEGs. Six upregulated genes and 15 downregulated genes were found in the merging dataset.

**FIGURE 3:**
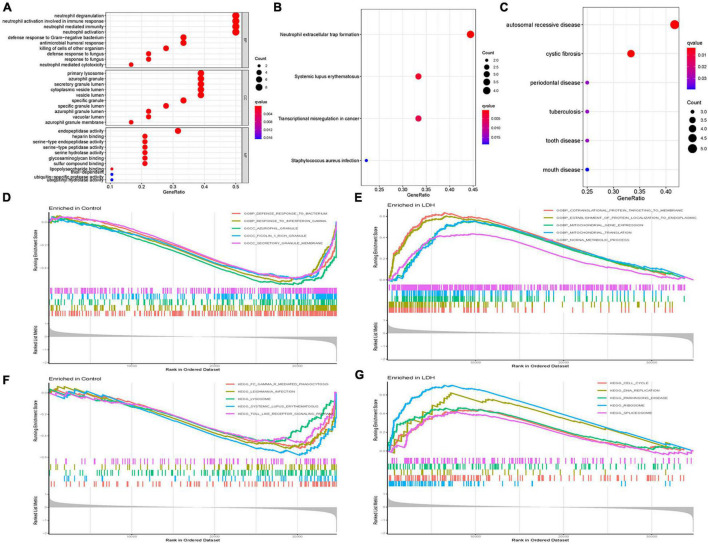
Functional correlation analysis. **(A)** Gene Ontology (GO) enrichment analysis, where the circle size represents the count of DEGs (the larger the circle size, the more count of DEGs), and the color represents *p*-value (the redder the color, the smaller the value). GO analyses consisted of biological process (BP), molecular function (MF), and cellular component (CC). **(B)** Kyoto Encyclopedia of Genes and Genomes (KEGG) enrichment analysis, where the circle size represents the count of DEGs, and the color represents *p*-value. **(C)** Disease Ontology (DO) enrichment analysis, where the circle size represents the count of DEGs, and the color represents *p*-value. **(D)** Gene Set Enrichment Analysis (GSEA) in the normal group using annotation information of GO. **(E)** GSEA in the LDH group using annotation information of GO. **(F)** GSEA in the normal group using annotation information of KEGG. **(G)** GSEA in the LDH group using annotation information of KEGG [Net enrichment score (NES), gene ratio, and *p*-value in GSEA analysis were used to verify the GO and KEGG enrichment results].

Figure 3 illustrates the results of functional correlation analysis of GO, KEGG, and DO. The results of GO analysis (Figure 3A) suggest that DEGs are mainly involved in 3 cellular functions: BP, CC, and MF. The primary variations in BP are neutrophil degranulation, neutrophil activation in immune response, neutrophil-mediated immunity, and neutrophil activation. The main differences in CC were the primary lysosome, azurophil granule, secretory granule lumen, cytoplasmic vesicle lumen, and vesicle lumen. The most variation in MF was the endopeptidase activity. Figure 3B illustrates the results of the KEGG pathway analysis. It shows that DEGs were significantly enriched in neutrophil extracellular trap formation, systemic lupus erythematosus, transcriptional misregulation in cancer, and staphylococcus aureus infection. DO analysis (Figure 3C) revealed that DEGs were primarily related to autosomal recessive disease, cystic fibrosis, periodontal disease, tuberculosis, tooth disease, and mouth disease.

Gene Set Enrichment Analysis (GSEA) and GO analysis (Figures 3D,E) showed that azurophil granules were significantly different between the healthy and LDH groups. The GSEA enrichment results showed the top five significant results in the healthy and LDH group. In addition, GSEA and KEGG analysis (Figures 3F,G) indicated that systemic lupus erythematosus was significantly different between the healthy and LDH groups.

### Screening and Verifying Diagnostic Biomarkers

**FIGURE 4:**
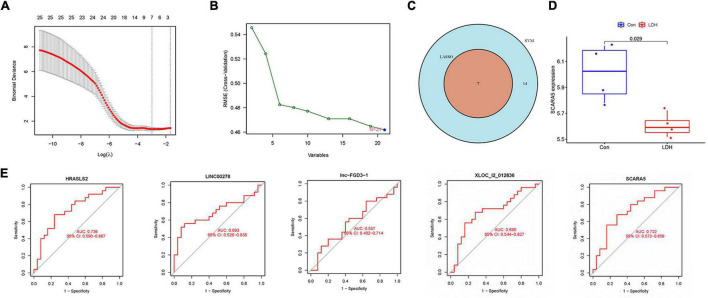
Identification of diagnostic biomarkers in LDH. **(A)** The gene numbers screened by the LASSO. **(B)** The gene numbers screened by the support vector machine recursive feature elimination (SVM-RFE). **(C)** The Venn plot was adopted to show the intersection genes between LASSO and SVM-RFE. **(D)** The distribution of scavenger receptor class A member 5 (SCARA5) between healthy and LDH group in GSE42611 dataset. **(E)** Receiver operating characteristic (ROC) curves showed the prediction efficiency of diagnostic markers.

Figure 4A illustrates gene numbers after using LASSO logistic regression algorithm to screen genes. Figure 4B presents the gene numbers after using SVM-RFE to screen genes. The ROC curves (Figure 4E) indicated the accuracy of five diagnostic biomarkers distinguishing the healthy and LDH patients, including XLOC_l2_012836 (AUC = 0.690), HRASLS2 (AUC = 0.736), scavenger receptor class A member 5 (SCARA5; AUC = 0.722), LINC00278 (AUC = 0.693), and lnc-FGD3-1 (AUC = 0.557). Figure 4C presents the seven intersection genes screened by both the LASSO and SVM-RFE methods. According to the result of the gene expression levels of three intersection genes in the GSE42611 dataset, the expression of SCARA5 was different in the nucleus pulposus (Figure 4D), while the expressions of Bactericidal permeability-increasing protein (BPI) and Cathepsin G (CTSG) were not significantly different.

**FIGURE 5:**
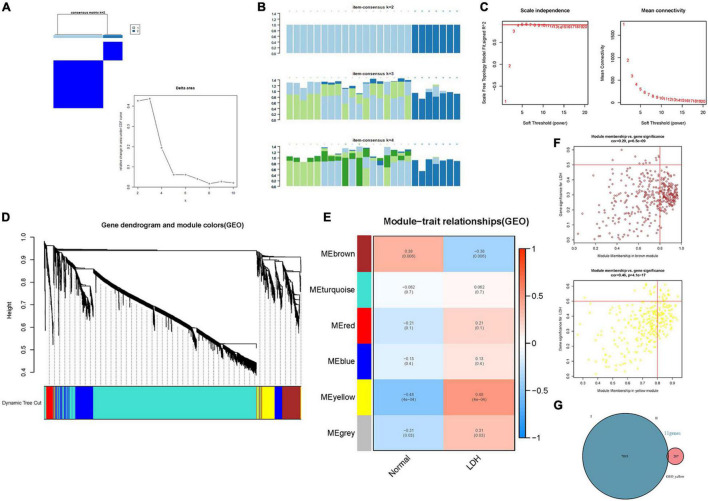
Identification of the hub genes in LDH. **(A)** Consensus clustering matrix for k = 2 in LDH dataset [Consensus clustering cumulative distribution function (CDF) for k = 2 to 9]; **(B)** Consensus clustering matrix (Each bar represents a grid, where the more complete the bar color, the better the clustering degree); **(C)** β = 4 (scale free *R*^2^ = 0.9) was chosen to construct a scale-free network; **(D)** A cluster tree of co-representation network modules based on 1-Tom matrix is constructed. **(E)** Module-trait relationships, where different colors represent different modules in two groups (each row corresponds to a module, and each unit includes a correlation coefficient and a *p*-value). **(F)** Module-gene relationships: each plot includes a correlation coefficient and a *p*-value. **(G)** Venn plot: identification of intersection genes between cluster genes and module genes.

### Identifying the Hub Genes

**FIGURE 6:**
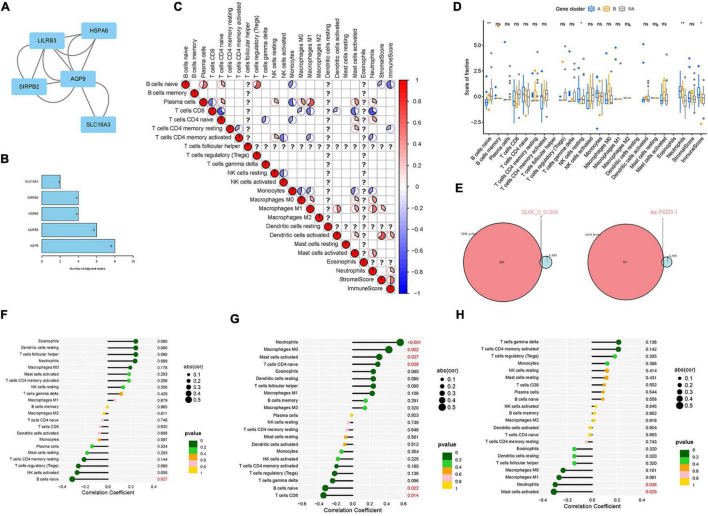
Identification of the hub genes in LDH and verification of the correlation between diagnostic biomarkers and immune cell infiltration. **(A)** The protein-protein interaction (PPI) network was constructed to identify the hub genes; **(B)** The number of adjacent nodes correlated with protein of hub genes; **(C)** analysis of the relationship between 22 types of immune cells of DEGs in LDH; **(D)** box plot: identification of significant immune cells between normal groups and LDH group (symbol “*,” “**,” and “ns,” respectively, stand for *p*-value under.05, *p*-value under.01, and non-significance.); **(E)** Venn plot: identification of intersection genes between biomarkers and module genes; **(F)** Expression level correlation between lnc-FGD3-1 and infiltrating immune cells; **(G)** Expression level correlation between XLOC_l2_012836 and infiltrating immune cells. **(H)** Expression level correlation between SCARA5 and infiltrating immune cells (The larger the circle size, the stronger the correlation; The color of the dots represents the *p*-value, and *p* < 0.05 was considered statistically significant).

Figure 5 illustrates the results of the WGCNA analysis. Within the six of the merged modules, two gene modules were significantly associated with LDH. Among them, the yellow module is positively correlated with LDH, and the brown module is negatively correlated with LDH. The yellow module consists of 287 genes, while the brown module consists of 384 genes. Venn plot was constructed to show the intersection genes between the two clustering groups (Figure 5G) and the two modules. Subsequently, 11 intersection genes were obtained between the yellow module and the clustering genes (Figure 5G), including ADCY4, AQP9, ATG16L2, ECEL1P2, HSPA6, LILRB3, lnc-F8A2-2, LOC101928948 (lncRNA), LOC729040 (lncRNA), SIRPB2, and SLC16A3. Figures 6A,B illustrate the results of the PPI network of intersection genes. As a result, five significant hub genes were identified (Figures 6A,B), namely, AQP9, LILRB3, HSPA6, SIRPB2, and SLC16A3. The ADCY4, AQP9, ECEL1P2, HSPA6, LILRB3, lnc-F8A2-2, LOC101928948, LOC729040, SIRPB2, and SLC16A3, all of which were found to have upregulated gene expressions. On the other hand, ATG16L2 was found to have downregulated gene expression in LDH.

### Verifying the Correlation of Biomarkers and Immune Cell Infiltration

The relationships between the 22 types of immune cells of DEGs in the LDH group were analyzed (Figure 6C). It was found that resting natural killer (NK) cells were positively related to plasma cells and CD4 memory-activated T cells. The heatmap revealed that neutrophils had a positive relation with plasma cells, CD4-naive T cells, macrophages M0, macrophages M1, and activated mast cells while having a negative correlation with CD8 T cells, CD4 memory-activated T cells, and monocytes. Then, the boxplot of immune infiltration (Figure 6D) revealed that naive B cells, resting NK cells, neutrophils, and immune scores have a significant difference between the healthy and LDH groups. Subsequently, Venn plots were applied to identify diagnostic markers and perform Spearman correlation analysis. Consequently, the lnc-FGD3-1 was identified when screening intersection genes in brown modules and genes obtained by LASSO logistic regression (Figure 6E). The XLOC_l2_012836 (lncRNA) was also identified when screening intersection genes in yellow modules and genes obtained by LASSO logistic regression (Figure 6E). Figures 6F–H illustrate the relationship between diagnostic biomarkers and immune cell infiltration. The expression level of lnc-FGD3-1 was negatively correlated with naive B cells (*r* = −0.313, *p* = 0.027) (Figure 6F). In addition, the expression level of XLOC_l2_012836 was positively correlated with neutrophils (*r* = 0.561, *p* < 0.001), macrophages M0 (*r* = 0.426, *p* = 0.002), activated mast cells (*r* = 0.313, *p* = 0.027), and CD4-naive T cells (*r* = 0.295, *p* = 0.038). The expression level of XLOC_l2_012836 was negatively related with naive B cells (*r* = −0.324, *p* = 0.022) and CD8 T cells (*r* = −0.346, *p* = 0.014) (Figure 6G). The expression level of SCARA5 was negatively correlated with neutrophils (*r* = −0.298, *p* = 0.036) and activated mast cells (*r* = −0.309, *p* = 0.029) (Figure 6H).

## Discussion

This study aimed to identify the vital diagnostic biomarkers and hub genes and to analyze the immune cell infiltration patterns in the LDH population. It is observed that the genes XLOC_l2_012836, lnc-FGD3-1, and SCARA5 correlated with the immune cell infiltration to various extents, which may, therefore, act as potential diagnostic biomarkers of LDH. Five hub genes were identified that correlated with LDH, including AQP9, SIRPB2, SLC16A3, LILRB3, and HSPA6. The new hub genes and immune infiltrating pattern identification extend the knowledge of the potential lumbar disc degeneration mechanisms.

In the present study, the GSEA and GO analysis (Figures 3D,E) showed that azurophil granule in the LDH and healthy group was significantly different. Azurophil granules released cytotoxic and digestive agents when neutrophils were guided to the site of infection (Cowland and Borregaard, 2016). The result supports the recent theory that inflammation plays a role in the cause of LDH. In addition, the GSEA and KEGG analysis (Figures 3F,G) indicated that the systemic lupus erythematosus pathway was significantly different between the LDH and the healthy group. A recent study suggested that NP cells could transform into fibroblast-like cells after the injury of the intervertebral disc (Au et al., 2020). Besides, a previous study found that systemic lupus erythematosus (SLE) was associated with the proliferation of fibrous tissue cells (Yamamoto et al., 2005). Though no study had provided firm evidence to support that SLE is correlated with the development of LDH, it is reasonable to speculate that SLE may contribute to the development of LDH by promoting the transformation of NP cells in the interverbal disc.

The LASSO logistic regression determines variables by looking for optional λ when the classification error is minimal. The SVM-RFE was used to achieve better performance by analyzing the appropriate dataset selected by the LASSO algorithm to obtain biomarkers. The intersection genes from the two modules of WGCNA and LASSO logistic regression identified significant differences in lnc-FGD3-1 and XLOC_l2_012836 between the LDH and healthy groups (Figure 6E). A long non-coding RNA (lncRNA) was found to play a vital role in the development of LDH by regulating cell proliferation and metastasis (Tang et al., 2020). Faciogenital dysplasia 3 (FGD3) has a presumed guanine nucleotide exchange factor which plays an important role in cell migration (Hayakawa et al., 2008). The FDG3 is found in the growth plate cartilage of the femurs of mice, which is associated with articular cartilage and growth plates (Takasuga et al., 2015). Therefore, it is reasonable to speculate that lnc-FGD3-1 may promote the degeneration of the intervertebral disc by regulating FDG3 expression and inhibiting the growth of the cartilage endplate. In addition, XLOC_l2_012836 (lncRNA) was positively correlated with neutrophils, M0 macrophages, activated mast cells, and CD4-naive T cells, while negatively correlated with naive B cells and CD8 T cells (Figure 6G). A previous study (Peng et al., 2006) suggested that macrophages and mast cells may play a vital role in repairing damaged AF and subsequent disc degeneration. This is given some support by a study that reported that imbalanced counts of CD4 + T and CD4 + /CD8 + lymphocytes were correlated with LDH-related back pain. Therefore, it could be proposed that XLOC_l2_012836 promotes the development of LDH by stimulating neutrophils, M0 macrophages, activated mast cells, and CD4-naive T cells, reducing naive B cells, and CD8 T cells. It is reasonable to speculate that XLOC_l2_012836 might play a vital role in the immune response in LDH. It is also reasonable to take SCARA5 into a relationship analysis between diagnostic biomarkers and immune cells after using the external validation of the GSE42611 dataset. The expression level of SCARA5 was negatively correlated with the expression of neutrophils (*r* = −0.298, *p* = 0.036) and activated mast cells (*r* = −0.309, *p* = 0.029) (Figure 6H). A previous study found that the downregulation expression of SCARA5 is correlated with the proliferation of synovial (de Seny et al., 2021) and cancer cells (Yan et al., 2012). From the result of the present study, the downregulated expression of SCARA5 might promote the proliferation of the nucleus pulposus to some extent and correlate with the occurrence of inflammation. More studies are needed to further analyze the function of lncRNA.

The hub genes of AQP9, SIRPB2, SLC16A3, LILRB3, and HSPA6 were significantly different between the LDH and healthy groups (Figures 6A,B). Aquaporin-9 (AQP9) is a hydroglycerin channel protein that promotes water movement between cerebrospinal fluid and brain parenchyma (Badaut et al., 2001). A study showed that AQP9 might be involved in chronic inflammation disease (Mesko et al., 2010). The downregulation of AQP9 was observed in the cartilage cells, which would cause the decomposition-related genes of stimulating the IL-1β that is down-expressed in osteoarthritis (Piccio et al., 2005). It is reasonable to suspect that the increased expression of AQP9 would promote the inflammation of CEP in the intervertebral disc, which subsequently contributes to LDH. Another potential theory is that owing to the lack of local blood supply, the NP cells settle in hypoxic conditions, which, thereby produces an increased amount of lactic acid and promotes the high expression of AQP9 (Badaut et al., 2001). Signal-regulatory protein beta 2 (SIRPB2), a transmembrane glycoprotein, is found to be expressed in the immune and central nervous system (Piccio et al., 2005). A recent study showed that the CD47 on antigen-presenting cells that engage with SIRPB2 on T cells could promote the proliferation of antigen-specific T-cells (Veillette and Chen, 2018). Therefore, the SIRPB2 might play a significant role in the immune response. The solute carrier family 16 member 3 (SLC16A3, also called MCT4), which is mainly affected by HIF-1α in NP cells at hypoxic conditions, plays a significant role in maintaining the stability of the intervertebral disc (Silagi et al., 2020). It is possible that due to the lack of blood supply, prolonged hypoxia could stimulate the increased expression of SLC16A3 and induce NP cell death. Leukocyte immunoglobulin-like receptor subfamily B member 3 (LILRB3, also called PIR-B) is found to be associated with the neutrophil activation and antibacterial effect function (Zhao et al., 2020). It might be reasonable to regard LILRB3 as an immune-induced treatment point since it can effectively inhibit immune response *in vitro* (Yeboah et al., 2020). The heat shock 70 kDa protein 6 (HSPA6) involves cell repairment and cell protection (Hageman et al., 2011). Becirovic and Brown (2017) illustrated that HSPA6 is involved in the post-stress transcriptional recovery in neurodegenerative diseases. Therefore, HSPA6 might be associated with the cell protection of LDH when facing stress. To date, there is no conclusive evidence confirming the above hub genes have the same pathological mechanism that contributes to the development of LDH. However, these genes are associated with the same factors, such as hypoxia or injury that promote the development of LDH. It might be a possible means to produce targeted therapy using these genes as the starting points.

## Limitations

In the present study, the cross-validation between LASSO logistic regression algorithm and SVM-RFE was applied to identify significant genes, followed by functional enrichment analysis to identify the mechanism. The data was then analyzed using the CIBERSORT method to explore the pattern of immune cells infiltration. However, there are still limitations about the effect of diagnostic markers in LDH. First, the present research is a retrospective study. Although external validation was added in the present study, there was no clinical trial to verify the accuracy of biomarker identification. Therefore, the functional impact of these RNAs in the occurrence and development of LDH ought to be assessed by knocking out or importing studies in animal models and cell lines. Second, the present study is a secondary analysis based on the originally published dataset. Although the results were broadly consistent with previous studies, the validity of the results should be examined with reasonable doubt. Moreover, the effect of treatment on the expression of RNA was not appraised. Despite the two chosen datasets being from the same research institute to minimize the error, the small sample size may contain bias.

## Conclusion

The present study identifies XLOC_l2_012836 (lncRNA), lnc-FGD3-1, and SCARA5 as potential genes for target therapy points. Their involvement in the development of LDH are potentially related to the immune response or inhibiting growth of cartilage endplate. The five identified hub genes are associated with the same factors of hypoxia or injury. The azurophil granule and the SLE pathway are significantly different between the healthy group and the LDH group after gene enrichment analysis. The findings of the present study provide some guidance for future research on the pathogenesis and treatment of LDH.

## Data Availability Statement

Publicly available datasets were analyzed in this study. These data can be found here: https://www.ncbi.nlm.nih.gov/geo/query/acc.cgi?acc=GSE124272; https://www.ncbi.nlm.nih.gov/geo/query/acc.cgi?acc=GSE150408; https://www.ncbi.nlm.nih.gov/geo/query/acc.cgi?acc=GSE42611.

## Author Contributions

KL and SL contributed to the literature search and design of the study protocol, drafted the manuscript, data collection, and data analysis. HZ and DL contributed to the data analysis and drafting of the manuscript. WL and MD managed the research and adjudicate any dispute, contributed to the funding acquisition and study management. All authors had read and approved the final manuscript, fulfilled the four authorship criteria, in addition, involved in a specific aspect of the study.

## Conflict of Interest

The authors declare that the research was conducted in the absence of any commercial or financial relationships that could be construed as a potential conflict of interest.

## Publisher’s Note

All claims expressed in this article are solely those of the authors and do not necessarily represent those of their affiliated organizations, or those of the publisher, the editors and the reviewers. Any product that may be evaluated in this article, or claim that may be made by its manufacturer, is not guaranteed or endorsed by the publisher.

## Footnotes

1
https://www.ncbi.nlm.nih.gov/geo/

2
https://www.r-project.org/

3
https://www.perl.org/get.html

4
https://string-db.org

## Abbreviations

LDH: Lumbar disc herniation
GEO: Gene Expression Omnibus
DEGs: Differentially Expressed Genes
LASSO: Least Absolute Shrinkage and Selection Operator
WGCNA: Weighted Gene Coexpression Network Analysis
LBP: Low back pain
AF: Annulus fibrous
NP: Nucleus pulposus
BNB: Blood-NP barrier
GO: Gene Ontology
KEGG: Kyoto Encyclopedia of Genes and Genomes
DO: Disease Ontology
BP: Biological process
MF: Molecular function
CC: Cellular component
FDR: False discovery rate
GSEA: Gene Set Enrichment Analysis
ROC: Receiver operating characteristic
TOM: Topological Overlap Matrix
GS: Gene significance
MM: Module membership
PPI: Protein-protein interaction
ICCs: Infiltrating immune cells
BPI: Bactericidal permeability-increasing protein
HRASLS: The H-RAS -like suppressor
CTSG: Cathepsin G
AQP9: Aquaporin 9
SIRPB2: Signal Regulatory Protein Beta 2
LILRB3: Leukocyte immunoglobulin-like receptor subfamily B member 3
HSPA6: Heat Shock Protein 6.
